# Use of Japanese Encephalitis Vaccine in Children: Recommendations of the Advisory Committee on Immunization Practices, 2013

**Published:** 2013-11-15

**Authors:** Joseph A. Bocchini, Lorry Rubin, Marc Fischer, Susan L. Hills, J. Erin Staples

**Affiliations:** Advisory Committee on Immunization Practices; Div of Vector-Borne Diseases, National Center for Emerging and Zoonotic Infectious Diseases, CDC

On June 19, 2013, the Advisory Committee on Immunization Practices (ACIP) voted to extend existing recommendations for use of inactivated Vero cell culture-derived Japanese encephalitis (JE) vaccine (JE-VC) (Ixiaro, Intercell Biomedical) to include children aged 2 months through 16 years (1). The ACIP JE Vaccine Workgroup reviewed the epidemiology of JE in travelers and evaluated published and unpublished data on JE-VC immunogenicity and safety in adults and children. The evidence for benefits and risks associated with JE-VC vaccination of children was evaluated using the Grading of Recommendations, Assessment, Development, and Evaluation (GRADE) framework (2,3). This report summarizes the evidence considered by ACIP and outlines the recommendations for use of JE-VC in children traveling to JE-endemic countries.

## JE Epidemiology and Risk for Disease in Travelers

JE virus, a mosquito-borne flavivirus, is an important cause of encephalitis in Asia (4). JE is a severe disease with a case fatality rate of 20%–30% and neurologic or psychiatric sequelae in 30%–50% of survivors (4). Although no specific treatment is available, the disease is vaccine-preventable.

The risk for JE for most travelers to Asia is very low, but varies based on destination, duration, season, and activities (4,5). The overall incidence of JE among persons from nonendemic countries traveling to Asia is estimated to be less than one case per 1 million travelers. However, the risk for JE among expatriates and travelers who stay for prolonged periods in rural areas with active JE virus transmission might be similar to the risk among the susceptible resident population (5–50 cases per 100,000 children per year) (4). Recurrent travelers or travelers on brief trips might be at increased risk if they have extensive outdoor or nighttime exposure in rural areas during periods of active transmission. Short-term travelers whose visits are restricted to major urban areas are at minimal risk for JE.

## JE Vaccine in the United States

JE-VC is the only JE vaccine licensed and available in the United States. An inactivated mouse brain–derived vaccine (JE-MB [JE-VAX]) previously was available and recommended for use in adults and children aged ≥1 year but is no longer being produced. In 2009, JE-VC was licensed and recommended for use in persons aged ≥17 years (4). In May 2013, the Food and Drug Administration (FDA) licensed JE-VC for use in children aged 2 months through 16 years (6).

## Dosage, Administration, and Schedule

The primary series for JE-VC is 2 intramuscular doses administered 28 days apart. For children aged 2 months through 2 years, each dose is 0.25 mL, and for adults and children aged ≥3 years, each dose is 0.5 mL. For persons aged ≥17 years, ACIP recommends that if the primary series of JE-VC was administered >1 year previously, a booster dose may be given before potential JE virus exposure (7). Although studies are being conducted on the need for a booster dose following a primary series of JE-VC in children, data are not yet available.

## JE-VC Licensure and Usage in Adults

No efficacy data are available for JE-VC. However, a JE virus 50% plaque reduction neutralization test (PRNT_50_) titer of ≥10 is an accepted immunologic correlate of protection (8,9). JE-VC was licensed based on its ability to induce seroprotective JE virus neutralizing antibody titers, a noninferiority comparison of safety and immunogenicity with JE-MB, and safety evaluations in approximately 5,000 adults (10–12). Since JE-VC was licensed in 2009, approximately 375,000 doses have been distributed in the United States for use in adults and no safety concerns have been identified (13,14).

## JE-VC Immunogenicity in Children

The pivotal pediatric clinical trial of JE-VC was conducted in children aged 2 months through 17 years in the Philippines (3,15,16). Among children randomly assigned to receive 2 age-appropriate doses of JE-VC, 384 (100%) of 385 were seroprotected at 28 days after the second dose (95% confidence interval [CI] = 96%–100%) (Table). At 6 months after completing the primary series, 134 (88%; CI = 82%–92%) of 152 children aged 2 months through 2 years and 224 (95%; CI = 91%–97%) of 237 children aged 3–17 years had protective neutralizing antibodies.

In a randomized, controlled trial conducted in India among children aged 1 and 2 years, 22 (96%; CI = 87%–100%) of 23 children were seroprotected at 28 days after receiving two 0.25 mL doses of JE-VC (3,15–17). No statistically significant differences were detected in the seroprotection rates between this group and children who received two 0.5 mL doses of JE-VC (20/21; 95%) (CI = 86%–100%) or 3 doses of an inactivated mouse brain–derived JE vaccine produced by the Korean Green Cross (10/11, 91%) (CI = 74%–100%).

In an observational study of children from nonendemic countries, all 51 children in the interim analysis had protective neutralizing antibodies at 28 days after the second dose of JE-VC (Table) (3,15,16). All 18 children evaluated remained seroprotected at 6 months after completing the primary series.

## JE-VC Safety Data for Children

In the open-label trial in the Philippines, 195 infants aged 2–11 months were randomly assigned to receive JE-VC (N = 131) or 7-valent pneumococcal conjugate vaccine (N = 64). An additional 1,674 children, aged 1–17 years, were randomly assigned to receive JE-VC (N = 1,280) or hepatitis A vaccine (N = 394) (3,15,16). The incidences of local, systemic, medically attended, and FDA-defined serious adverse events were similar between children who received JE-VC or the comparison vaccines. Overall, 9% (122/1,411) of JE-VC recipients had fever (≥100.4°F [≥38.0°C]) within 7 days after the first dose and 6% (84/1,405) had fever within 7 days after the second dose. Within 1 month after either dose, four (<1%) recipients had urticaria or hypersensitivity reactions, and five (<1%) had neurologic adverse events, including febrile seizures (N = 3), drooling (N = 1), and dizziness (N = 1); all were similar to rates for recipients of the comparison vaccines. Among the 1,411 children who received JE-VC, 23 (2%) reported a serious adverse event within 7 months of the first dose. The most common serious adverse events were pneumonia (N = 6) and febrile seizures (N = 5). Only three serious adverse events were reported within 2 weeks after a dose of JE-VC, including one report each of a febrile convulsion, cellulitis, and gastroenteritis. One death resulted from suspected bacterial meningitis and pneumonia in a male aged 12 years at 4 months after the second dose of JE-VC. No other neurologic or hypersensitivity events were reported as serious adverse events.

Among the 48 children aged 1 and 2 years who were randomly assigned to receive JE-VC in India, five (10%) reported injection site tenderness, and one (2%) reported fever within 7 days after either dose (3,15–17). The only unsolicited adverse events were one report each of skin lesion and skin rash. No serious adverse events or deaths were reported.

In the observational study of children aged 2 months through 17 years from nonendemic countries, among 60 children included in the interim analysis, four (7%) had fever, 22 (37%) had injection site tenderness, and 15 (25%) had muscle pain in the 7 days after either JE-VC dose (3,15,16). Two serious adverse events were reported, one child each with diabetes mellitus (3 months after dose 2) and dizziness (4 months after dose 2). No other neurologic or hypersensitivity adverse events were reported.

## Rationale for JE Vaccine Recommendations

Considerations in providing recommendations for use of JE-VC in travelers include 1) the overall low risk for travel-associated JE, which varies based on itinerary and activities, 2) the lack of available treatment and high rates of morbidity and mortality when the disease does occur, and 3) the high rates of seroprotection and low probability of serious adverse events following vaccination (3,4,14). Travel vaccines are usually paid for by the travelers themselves; they are not covered under the Vaccines for Children (VFC) program or by most private insurance plans. A cost-effectiveness study of JE vaccine for U.S. children traveling to JE-endemic countries was not performed. However, given the large numbers of travelers to Asia (>5.5 million U.S. travelers entered JE-endemic countries in 2004), the low risk for JE for most travelers to Asia, and the high cost of JE-VC ($400–$500 per 2-dose primary series), providing JE vaccine to all travelers to Asia likely would not be cost-effective. In addition, for some travelers with lower risk itineraries, even a low probability of vaccine-related serious adverse events might be higher than the risk for disease. Therefore, JE vaccine should be targeted to travelers who, on the basis of their planned travel itinerary and activities, are at higher risk for disease (Box) (4).

## ACIP Recommendations for Use of JE-VC in Children

ACIP recommendations for use of JE-VC for the primary series in children aged 2 months through 16 years are the same as for persons aged ≥17 years (Box) (4). Travelers to JE-endemic countries should be advised of the risks for JE disease and the importance of personal protective measures to reduce the risk for mosquito bites. For some travelers who will be in a higher-risk setting based on season, location, duration, and activities, JE vaccine can further reduce the risk for infection. JE vaccine is recommended for travelers who plan to spend a month or longer in endemic areas during the JE virus transmission season. JE vaccine should be considered for short-term (<1 month) travelers whose itinerary or activities might increase their risk for exposure to JE virus. JE vaccine is not recommended for short-term travelers whose visit will be restricted to urban areas.

BOX Recommendations for use of inactivated Vero cell culture–derived Japanese encephalitis (JE) vaccine in adults and children aged ≥2 months traveling to JE-endemic areas*

- JE vaccine is recommended for travelers who plan to spend a month or longer in endemic areas during the JE virus transmission season. This includes long-term travelers, recurrent travelers, or expatriates who will be based in urban areas but are likely to visit endemic rural or agricultural areas during a high-risk period of JE virus transmission.
- JE vaccine should be considered for the following persons:
  – Short-term (<1 month) travelers to endemic areas during the JE virus transmission season if they plan to travel outside of an urban area and have an increased risk for JE virus exposure (e.g., spending substantial time outdoors in rural or agricultural areas, participating in extensive outdoor activities, staying in accommodations without air conditioning, screens, or bed nets).
  – Travelers to an area with an ongoing JE outbreak.
  – Travelers to endemic areas who are uncertain of specific destinations, activities, or duration of travel.
- JE vaccine is not recommended for short-term travelers whose visit will be restricted to urban areas or periods outside of a well-defined JE virus transmission season.

## Figures and Tables

**TABLE:** Seroprotection (PRNT_50_ titer ≥10) at 1 month after a 2-dose primary series of inactivated Vero cell culture–derived Japanese encephalitis vaccine (JE-VC) administered according to the dose and schedule approved by the Food and Drug Administration (FDA)*

		0.25 mL dose		0.5 mL dose	
			
Study location	Age group	No.	(%)	No.	(%)
Philippines	2 mos–17 yrs	147/148	(99)†	237/237	(100)
India	1–2 yrs	22/23	(96)	—	—§
United States/Europe/Australia	2 mos–17 yrs	5/5	(100)	46/46	(100)

**Abbreviation:** PRNT_50_ = 50% plaque reduction neutralization test.

* For children aged 2 months–2 years, two 0.25 mL doses administered 28 days apart; for children aged 3–17 years, two 0.5 mL doses administered 28 days apart.

† Of an additional 98 children aged 3–11 years who received two 0.25 mL doses, 94 (96%) were seroprotected at 1 month after the second dose.

§ Of 21 children aged 1–2 years who received two 0.5 mL doses, 20 (95%) were seroprotected at 1 month after the second dose; the FDA-approved dose for children aged 1–2 years is 0.25 mL.

## Footnotes

* Information on JE virus transmission by country is available from CDC at http://wwwnc.cdc.gov/travel/yellowbook/2014/chapter-3-infectious-diseases-related-to-travel/japanese-encephalitis.
